# Precursor Epithelial Subtypes of Adenocarcinoma Arising from Intraductal Papillary Mucinous Neoplasms (A-IPMN): Clinicopathological Features, Recurrence and Response to Adjuvant Chemotherapy

**DOI:** 10.1245/s10434-024-15677-z

**Published:** 2024-07-03

**Authors:** James Lucocq, Beate Haugk, Daniel Parkinson, Antony Darne, Nejo Joseph, Jake Hawkyard, Steve White, Omar Mownah, Krishna Menon, Takaki Furukawa, Yosuke Inoue, Yuki Hirose, Naoki Sasahira, Anubhav Mittal, Jas Samra, Amy Sheen, Michael Feretis, Anita Balakrishnan, Carlo Ceresa, Brian Davidson, Rupaly Pande, Bobby V. M. Dasari, Lulu Tanno, Dimitrios Karavias, Jack Helliwell, Alistair Young, Quentin Nunes, Tomas Urbonas, Michael Silva, Alex Gordon-Weeks, Jenifer Barrie, Dhanny Gomez, Stijn van Laarhoven, Hossam Nawara, Joseph Doyle, Ricky Bhogal, Ewen Harrison, Marcus Roalso, Deborah Ciprani, Somaiah Aroori, Bathiya Ratnayake, Jonathan Koea, Gabriele Capurso, Ruben Bellotti, Stefan Stättner, Tareq Alsaoudi, Neil Bhardwaj, Fraser Jeffery, Saxon Connor, Andrew Cameron, Nigel Jamieson, Keith Roberts, Kjetil Soreide, Anthony J. Gill, Sanjay Pandanaboyana

**Affiliations:** 1 Department of General Surgery, NHS Lothian, Edinburgh, UK; 2 Hepatopancreatobiliary and Transplant Unit, Freeman Hospital, Newcastle Upon Tyne, UK; 3 Department of Hepatobiliary and Pancreatic Surgery, King’s College Hospital, Denmark Hill, London, UK; 4 Hepato-Biliary-Pancreatic Medicine Department, Cancer Institute Hospital of Japanese Foundation for Cancer Research, Tokyo, Japan; 5 Royal North Shore Hospital, Sydney, NSW Australia; 6 Department of Anatomical Pathology, Royal North Shore Hospital, New South Wales Health Pathology, Sydney, NSW Australia; 7 Cambridge Hepatobiliary and Pancreatic Surgery Unit, Addenbrooke’s Hospital, Cambridge, UK; 8 Hepatobiliary and Pancreatic Surgery Unit, The Royal Free Hospital, London, UK; 9 Hepatobiliary and Pancreatic Surgery Unit, University Hospitals Birmingham NHS Foundation Trust, Queen Elizabeth Hospital Birmingham, Birmingham, UK; 10 Hepatobiliary and Pancreatic Surgery Unit, University Hospital Southampton, Southampton, UK; 11 Hepatobiliary and Pancreatic Surgery Unit, Leeds Teaching Hospitals NHS Trust, Leeds, UK; 12 Department of Hepatopancreatobiliary Surgery, East Lancashire Teaching Hospitals NHS Trust, Lancashire, UK; 13 Oxford Hepato-Pancreato-Biliary Surgical Unit, Oxford University Hospitals NHS Foundation Trust, Lancashire, UK; 14 Nottingham Hepato-Pancreatico-Biliary Service, Nottingham University Hospitals NHS Foundation Trust, Lancashire, UK; 15 Department of Hepatobiliary and Pancreatic Surgery, University Hospitals Bristol & Weston NHS Foundation Trust, Bristol, UK; 16 Gastrointestinal Unit, The Royal Marsden NHS Foundation Trust, London, UK; 17 Department of Clinical Surgery, University of Edinburgh, Edinburgh, UK; 18 Department of Gastrointestinal Surgery, Stavanger University Hospital, Stavanger, Norway; 19 Hepatopancreatobiliary Unit, University Hospitals Plymouth NHS Trust, Plymouth, UK; 20 Hepato-Pancreatico-Biliary/Upper Gastrointestinal Unit, North Shore Hospital, Auckland, New Zealand; 21 Pancreatico-Biliary Endoscopy and Endosonography Division, Pancreas Translational and Clinical Research Centre, San Raffaele Scientific Institute IRCCS, Vita-Salute San Raffaele University, Milan, Italy; 22 Department of Visceral, Transplant and Thoracic Surgery, Centre of Operative Medicine, Medical University of Innsbrusk, Innsbruck, Austria; 23 Leicester Hepatopancreatobiliary Unit, University Hospitals of Leicester NHS Trust, Leicester, UK; 24 Department of General and Vascular Surgery, Christchurch Hospital, Canterbury District Health Board, Christchurch, New Zealand; 25 Wolfson Wohl Cancer Research Centre, Research Institute of Cancer Sciences, University of Glasgow, Glasgow, UK; 26 Sydney Medical School, University of Sydney, Sydney, NSW Australia

**Keywords:** IPMN, Adjuvant chemotherapy, Epithelial subtypes, Recurrence, Survival, Pancreatobiliary, Gastric, Intestinal

## Abstract

**Background:**

The clinico-oncological outcomes of precursor epithelial subtypes of adenocarcinoma arising from intraductal papillary mucinous neoplasms (A-IPMN) are limited to small cohort studies. Differences in recurrence patterns and response to adjuvant chemotherapy between A-IPMN subtypes are unknown.

**Methods:**

Clincopathological features, recurrence patterns and long-term outcomes of patients undergoing pancreatic resection (2010–2020) for A-IPMN were reported from 18 academic pancreatic centres worldwide. Precursor epithelial subtype groups were compared using uni- and multivariate analysis.

**Results:**

In total, 297 patients were included (median age, 70 years; male, 78.9%), including 54 (18.2%) gastric, 111 (37.3%) pancreatobiliary, 80 (26.9%) intestinal and 52 (17.5%) mixed subtypes. Gastric, pancreaticobiliary and mixed subtypes had comparable clinicopathological features, yet the outcomes were significantly less favourable than the intestinal subtype. The median time to recurrence in gastric, pancreatobiliary, intestinal and mixed subtypes were 32, 30, 61 and 33 months. Gastric and pancreatobiliary subtypes had worse overall recurrence (*p* = 0.048 and *p* = 0.049, respectively) compared with the intestinal subtype but gastric and pancreatobiliary subtypes had comparable outcomes. Adjuvant chemotherapy was associated with improved survival in the pancreatobiliary subtype (*p* = 0.049) but not gastric (*p* = 0.992), intestinal (*p* = 0.852) or mixed subtypes (*p* = 0.723). In multivariate survival analysis, adjuvant chemotherapy was associated with a lower likelihood of death in pancreatobiliary subtype, albeit with borderline significance [hazard ratio (HR) 0.56; 95% confidence interval (CI) 0.31–1.01; *p* = 0.058].

**Conclusions:**

Gastric, pancreatobiliary and mixed subtypes have comparable recurrence and survival outcomes, which are inferior to the more indolent intestinal subtype. Pancreatobiliary subtype may respond to adjuvant chemotherapy and further research is warranted to determine the most appropriate adjuvant chemotherapy regimens for each subtype.

Intraductal papillary mucinous neoplasms (IPMNs) are pancreatic precursor lesions for pancreatic adenocarcinoma; between 20% and 61% of IPMNs demonstrate malignant transformation in the surgical specimen.^1–3^ The precursor epithelial subtype of the IPMN (e.g. gastric, pancreatobiliary, intestinal and mixed) can indicate prognosis and likelihood of invasive disease, but its prognostic value in those with invasive disease is less clear.^4–7^ Our study group previously reported that recurrence following pancreatic resection for adenocarcinoma arising from intraductal papillary mucinous neoplasms (A- IPMN) is frequent, with a quarter of patients recurring within 12 months.^8^ To the best of our knowledge, whether epithelial subtype determines recurrence rates or patterns of recurrence in A-IPMN have not been previously investigated.

Adjuvant chemotherapy is currently advised by the European guidelines on pancreatic cystic tumours for A-IPMN regardless of nodal status, and may have survival benefit in patients with A-IPMN and node positive disease.^9,10^ Evidence for adjuvant chemotherapy in A-IPMN precursor epithelial subtypes and whether or not subtype influences chemotherapy response have not been investigated.^11–13^

The aim of the present study was to report clinicopathological associations, recurrence patterns, response to adjuvant chemotherapy and overall survival of precursor epithelial subtypes in patients with A-IPMN.

## Patients and Methods

### Study Characteristics

Patients who had undergone pancreatic resection between 2010 and December 2020 for A-IPMNs with an available precursor subtype were retrospectively identified from 18 academic pancreatic cancer centres in Europe, Asia, Australia and New Zealand. The methodology has previously been published by our research group where we investigated the impact of treatment of recurrence on survival.^8^ The presence of A-IPMN were identified retrospectively on the basis of histopathological specimens following resection. The institutional review board of each participating institution approved the study prior to initiation and was conducted according to the Declaration of Helsinki. The Research Electronic Data Capture (REDCAP) system was used to store anonymized information that was then maintained by the Newcastle Joint Research Office. Informed patient consent was not required given the retrospective nature of the study. The Strengthening the Reporting of Observational Studies in Epidemiology (STROBE) recommendations were followed and adhered to as best as possible.

### Preoperative Work-up and Surgical Indications

Endoscopic ultrasound (EUS) and either aspiration or biopsy with cytological or biochemical analyses were performed at the discretion of the participating institution. The type of surgical procedure [e.g. Whipples, pylorus preserving pancreatoduodenectomy (PPPD), distal pancreatectomy with splenectomy (DPS), distal pancreatectomy without splenectomy (DPNS) and total pancreatectomy (TP)] and the decision to administer adjuvant chemotherapy was at the discretion of each participating institution on the basis of the location, degree and extent of the tumour.^14^ In cases of borderline-resectable disease, a portal or superior mesenteric vein resection was performed if infiltration was suspected. Patients with metastatic disease or locally advanced disease were excluded.

### Histopathological Diagnosis

A-IPMNs were defined using the criteria outlined in the current World Health Organization (WHO) 2019 classification—that is unequivocal invasive growth of a malignancy arising from IPMNs.^15^ Intra-ductal oncolytic papillary neoplasms (IOPNs) were previously considered a epithelial subtype of IPMNs, as per the 2010 WHO classification; however, under the fifth edition of the WHO classification of tumours published in 2019, IOPNs have been considered a distinct and separate entity to IPMNs. This update is primarily based on morphological and genetic differences, and as such invasive IOPN were excluded (*n* = 20).^15–17^

Cases where a synchronous pancreatic ductal adenocarcinoma (PDAC) was identified but clearly developed away from an IPMN (PDAC with concomitant IPMN), were excluded. The invasive component of the IPMN tumours were classified as either ductal or colloid on the basis of the criteria described in the WHO 2019 system: colloid carcinomas were defined as adenocarcinomas in which ≥ 80% of the neoplastic epithelium are suspended in extracellular mucin pools.^15,16,18^ All tumours were staged according to American Joint Committee on Cancer (AJCC) Staging System, eighth editiion.^18^

The precursor epithelial type of each lesion was determined using WHO 2019 criteria.^15^ In cases where the underlying precursor epithelial lesion or subtype were not reported in the original reporting, the pathology slides were reviewed again to report these findings. Where IPMNs had more than one epithelial subtype the epithelial subtypes were reported, and these patients were grouped as ‘mixed A-IPMN’. Margins were assessed using a structured/synoptic format, and for the purposes of determination of the R status, R1 margins were based on a cut-off distance from the tumour to the resection margin of < 1 mm.^18^ The IPMN duct-type was classified on pathology as main duct, branch duct or mixed type, according to the consensus guidelines.^14,18^

### Statistical Analyses

The clinicopathological features of A-IPMNs were compared with Chi-squared and Fischer’s exact test for binary variables. Non-parametric continuous variables were compared with the Mann–Whitney *U* test.

Recurrence patterns, including locoregional and systemic recurrence, were reported for each subtype, and median time to recurrence was determined using Kaplan–Meier (KM) analysis. The logrank test was used to compare between subgroups. All statistical analyses were performed in R studio, 2022.02.1 using the ‘survival’ package.

For each precursor epithelial subtype, the impact of adjuvant chemotherapy on overall recurrence and survival were assessed using the logrank test. Cox proportional hazards models (CPHMs) were also used to determine the effect of adjuvant chemotherapy on recurrence and survival for each precursor subtype after accounting for high-risk clinicopathological features: Charlson comorbidity index (CCI), AJCC stage, differentiation, perineural invasion, lymphovascular invasion and R1 resection. In CPHMs the dummy variables included low CCI, AJCC 1a/1b/2a, well/moderately differentiated, R0 resection and no peri-neural/lymphovascular invasion. Low CCI was considered any value below the median for the cohort. The effect of adjuvant chemotherapy type [e.g. gemcitabine (GEM), gemcitabine-capecitabine (GEM-CAP) and FOLFIRINOX (FFX)] on outcome was determined using for each subgroup.

## Results

In total, 297 patients were included (median age, 70 years; male, 78.9%) including 54 (18.2%) gastric, 111 (37.3%) pancreatobiliary, 80 (26.9%) intestinal and 52 (17.5%) mixed subtypes (Table 1). The 52 mixed lesions included 32 (61.5%) lesions with gastric subtype, 17 (32.7%) intestinal subtype and 43 (82.7%) pancreatobiliary subtype. There were five (9.6%) patients with an associated IOPN, without oncocytic invasion. The cohort was followed up for a median of 6 years and 5 months for recurrence and survival outcomes.

**Table 1:** Pre-operative characteristics of IPMN by epithelial precursor

Pre-operative characteristics		All (*n* = 297)	IPMN epithelial precursor subtype			
			Gastric (*n* = 54)	Pancreatic (*n* = 111)	Intestinal (*n* = 80)	Mixed (*n* = 52)
Median age (years)		70	68	70	68	72
Male (%)		239 (80.4)	25 (46.3)	64 (57.7)	51 (63.8)	28 (53.8)
Charlson comorbidity index, median (range)		4	4	5	4	5
Surveillance (%)		54 (18.2)	10 (18.5)	19 (17.1)	16 (20.0)	9 (17.3)
Borderline resectable (%)		16 (5.4)	2 (3.7)	6 (5.4)	4 (5.0)	4 (7.7)
Ca19-9 (U/ml) (%)	Median	41	24.5	87	32	38.5
	High	87 (32.2)	12/32 (37.5)	37/61 (60.7)	25/58 (43.1)	19/36 (52.8)
EUS (%)	Total	198 (66.9)	34 (63.0)	64 (57.7)	60 (75.0)	40 (76.9)
	Malignancy on needle biopsy	51/110 (46.4)	8/18 (44.4)	18/36 (50.0)	10/16 (62.5)	15/22 (68.2)
EUS aspiration (%)	Malignant	32/96 (33.3)	4/15 (26.7)	12/30 (40.0)	11/40 (27.5)	5/19 (26.3)
	Median CEA (ng/ml)	31	15	547	4.4	751
	Aspiration mucin	39/57 (68.4)	7/9 (77.8)	11/17 (64.7)	17/24 (70.8)	4/7 (57.1)
	Aspiration amylase	18/57 (31.6)	2/9 (22.2)	6/17 (35.3)	7/24 (29.2)	3/7 (42.9)
Duct type (%)	Main duct	183 (61.6)	33 (61.1)	63 (56.8)	62 (77.5)	25 (48.1)
	Branch	41 (13.8)	13 (24.1)	21 (18.9)	4 (5.0)	3 (5.8)
	Mixed	68 (22.9)	7 (13.0)	27 (24.3)	11 (13.75)	23 (44.2)
Location (%)	Head	186 (62.6)	33 (61.1)	69 (62.1)	53 (66.25)	31 (59.6)
	Body	28 (9.4)	4 (7.4)	8 (7.2)	11 (13.75)	5 (9.6)
	Tail	45 (15.2)	11 (20.4)	20 (18.0)	9 (11.25)	5 (9.6)
	Diffuse	37 (12.5)	6 (11.1)	13 (11.7)	7 (8.75)	11 (21.2)

### Clinicopathological Features

The clinicopathological characteristics (Table 1) and both operative and histopathological details (Table 2) of the precursor epithelial subtypes are reported. There were no statistically significant differences in clinicopathological, operative or histopathological features between mixed, gastric or pancreatobiliary subtypes.

**Table 2:** Operative and histopathological features by epithelial subtype

Variables	All (*n* = 297)	IPMN precursor epithelial subtype			
		Gastric (*n* = 54)	Pancreatic (*n* = 111)	Intestinal (*n* = 80)	Mixed (*n* = 52)
Operation (%)					
Whipples	104 (35.0)	19 (35.2)	40 (36.0)	36 (45.0)	9 (17.3)
PPPD	54 (182.)	13 (24.1)	18 (16.2)	9 (11.25)	14 (26.9)
DPS	53 (17.8)	14 (25.9)	21 (18.9)	12 (15.0)	6 (11.5)
DPNS	8 (2.7)	0 (0.0)	4 (3.6)	1 (1.25)	3 (5.8)
TP	78 (26.3)	8 (14.8)	28 (25.2)	22 (27.5)	20 (38.5)
Neoadjuvant chemotherapy (%)	17 (5.7)	3 (5.6)	4 (3.6)	7 (8.75)	3 (5.8)
Multivisceral resection (%)	44 (14.8)	7 (13.0)	16 (14.4)	12 (15.0)	9 (17.3)
Cyst size, median (IQR) mm (%)	30	30	31	35	30
Differentiation (%)					
Well	35 (11.8)	8 (14.8)	15 (13.5)	10 (12.5)	2 (3.8)
Moderately	170 (57.2)	25 (46.3)	67 (60.4)	50 (62.5)	28 (53.8)
Poor	79 (26.6)	20 (37.0)	27 (24.3)	13 (16.25)	19 (36.5)
Invasive components (%)					
Ductal	237 (79.8)	49 (90.7)	99 (89.2)	42 (52.5)	42 (80.8)
Colloid	47 (15.8)	5 (9.3)	7 (6.3)	28 (35.0)	7 (13.5)
Lymphovascular invasion (%)	152 (51.2)	28 (51.9)	64 (57.7)	33 (41.25)	27 (51.9)
Perineural invasion (%)	179 (60.2)	28 (51.9)	65 (58.6)	38 (47.5)	28 (53.8)
R1 (%)	124 (41.8)	21 (38.9)	47 (42.3)	33 (41.25)	23 (44.2)
N1 or N2 (%)	136 (45.7)	31 (57.4)	55 (49.5)	26 (32.5)	24 (46.2)
AJCC stage (%)					
1a	51 (17.2)	7 (12.9)	14 (12.6)	14 (17.5)	16 (30.8)
1b	38 (12.8)	6 (11.1)	12 (10.8)	15 (18.75)	5 (9.6)
2a	48 (16.2)	5 (9.3)	19 (17.1)	19 (23.75)	5 (9.6)
2b	132 (44.4)	28 (51.9)	56 (50.5)	25 (31.25)	23 (44.2)
3	28 (2.7)	8 (14.8)	10 (9.0)	7 (8.75)	3 (5.8)
Clavien–Dindo grade (%)					
CD 2	83 (27.9)	15 (27.8)	33 (29.7)	18 (22.5)	17 (32.7)
CD ≥ 3	51 (17.2)	11 (20.4)	16 (14.4)	20 (25.0)	4 (7.8)

In comparison with intestinal subtype, gastric subtypes had a preponderance towards female sex (53.7% versus 36.2%; *p* = 0.045), branch duct location (24.1% versus 5.0%; *p* = 0.009) and PPPD (24.1% versus 11.3% *p* = 0.045).

Gastric subtypes also had higher rates of ductal invasion (90.7% versus 52.5%; *p* < 0.001), poor differentiation (*p* = 0.006), positive lymph nodes (*p* = 0.004) and rates of AJCC stage 2b (51.9% versus 31.3%; *p* = 0.017).

Compared with intestinal subtype, pancreatobiliary subtype were more likely to arise from a branch duct (5.0% versus 18.9%; *p* = 0.005), have ductal invasion (89.2% versus 52.5%; *p* < 0.001), lymphovascular invasion (57.7% versus 41.3%; *p* = 0.025), lymph node involvement (49.5% versus 32.5%, *p* = 0.019) and be of AJCC Stage 2b (50.5% versus 31.3%; *p* = 0.008).

Mixed precursor epithelial subtype had a higher rate of mixed-duct location (44.2% versus 13.8%; *p* < 0.001), ductal invasion (80.8% versus 52.5%; *p* = 0.001) and rates of poor differentiation (36.5% versus 16.3%; *p* = 0.008) compared with intestinal subtype.

### Recurrence Outcomes

**Fig. 1:**
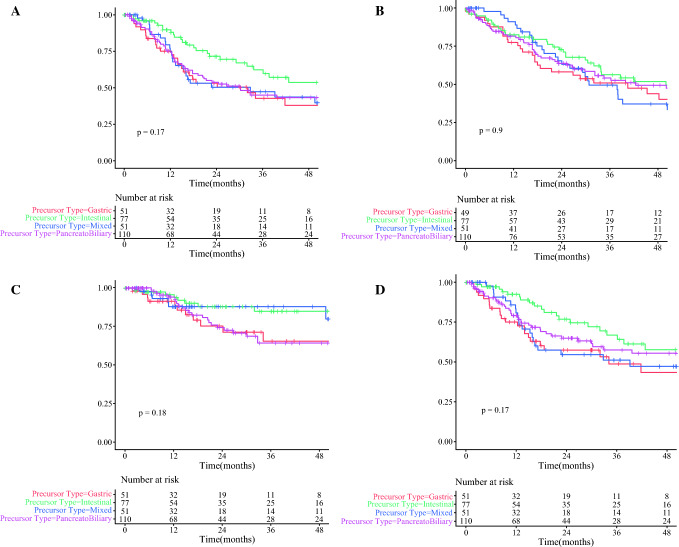
Kaplan–Meier curve for A-IPMN precursor epithelial subtypes illustrating rates of (**A**) recurrence, (**B**) overall survival, (**C**) locoregional recurrence and (**D**) systemic recurrence

The median time to recurrence in gastric, pancreatobiliary, intestinal and mixed subtypes was 32, 30, 61 and 33 months, respectively (Table 3, Fig. 1). Gastric, pancreatobiliary and mixed subtype had comparable overall recurrence rates (logrank, *p* = 0.813), but each were inferior to intestinal subtype (logrank test, *p* = 0.048, *p* = 0.049 and *p* = 0.082, respectively).

**Table 3:** Outcomes by epithelial subtype

Outcome variable	All (*n* = 297)	IPMN precursor epithelial subtype			
		Gastric (*n* = 54)	Pancreatic (*n* = 111)	Intestinal (*n* = 80)	Mixed (*n* = 52)
Adjuvant chemotherapy (%)					
Any	184 (61.9)	28 (51.8)	66 (59.5)	46 (57.5)	34 (65.4)
Gem	101 (34.0)	11 (20.4)	41 (36.9)	24 (30.0)	18 (34.6)
Gem-Cap	49 (16.5)	8 (14.8)	14 (12.6)	12 (15.0)	12 (23.1)
FFX	19 (6.4)	6 (11.1)	4 (3.6)	7 (8.75)	2 (3.8)
Other	15 (5.1)	3 (5.6)	7 (6.3)	0 (0.0)	2 (3.8)
Recurrence (%)					
All	130 (43.8	26 (48.1)	53 (47.7)	26 (32.5)	25 (48.1)
Locoregional	55 (18.5)	12 (22.2)	24 (21.6)	10 (12.5)	6 (11.5)
Systemic	108 (36.4)	22 (40.7)	39 (35.1)	20 (25.0)	21 (40.4)
Recurrence rate (%)					
1 year	56/295 (19.0)	12/54 (22.2)	25/110 (22.7)	8/80 (10.0)	11/51 (21.6)
2 year	103/281 (36.7)	22/53 (41.5)	43/104 (41.3)	17/75 (22.7)	21/49 (42.9)
5 year	125/190 (65.8)	25/33 (75.8)	51/76 (67.1)	25/52 (48.1)	24/29 (82.8)
10 year	29/53 (54.7)	6/13 (46.2)	10/18 (55.6)	10/17 (58.8)	3/5 (60.0)
Treatment for recurrence (%)					
Any	85 (28.6)	10 (18.5)	28 (25.2)	16 (20.0)	20 (38.5)
Chemo	61 (20.5)	9 (16.7)	26 (23.4)	10 (12.5)	9 (17.3)
Radio	16 (5.4)	0 (0.0)	2 (1.8)	7 (8.75)	6 11.5)
Surgery	6 (2.0)	1 (1.9)	2 (1.8)	2 (2.5)	1 (.9)
Secondary recurrence (%)	17 (5.7)	4 (7.4)	8 (7.2)	2 (2.5)	2 (3.8)
Death rate (%)					
1 year	47/295 (15.9)	11/54 (20.3)	19/110 (17.3)	13/80 (16.3)	4/51 (7.8)
2 year	90/281 (32.0)	20/53 (37.7)	35/104 (33.7)	19/75 (25.3)	16/49 (32.7)
5 year	137/190 (72.1)	26/33 (78.7)	49/76 (64.5)	35/52 (67.3)	27/29 (93.1)
10 year	36/53 (67.9)	7/13 (53.8)	11/18 (61.1)	12/17 (70.6)	4/5 (80.0)

Gastric subtype (median time to recurrence 34 months) had significantly higher rates of systemic recurrence than intestinal subtype (median time to recurrence, undefined; logrank, *p* = 0.032). There were no significant differences in rates of locoregional recurrence between subgroups. Table 4 reports the recurrence patterns for each precursor epithelial subtype.

**Table 4:** Site of recurrence in A-IPMNs

Site of recurrence	IPMN Precursor Epithelium			
	Gastric (*n* = 54)	Pancreatobiliary (*n* = 111)	Intestinal (*n* = 80)	Mixed (*n* = 52)
Locoregional (%)	12 (22.2)	24 (21.6)	10 (12.5)	6 (11.5)
Liver (%)	10 (18.5)	15 (13.5)	6 (7.5)	10 (19.2)
Lung (%)	5 (9.3)	19 (17.1)	5 (6.3)	7 (13.5)
Peritoneal (%)	9 (16.7)	8 (7.2)	9 (11.3)	5 (9.6)
Other (%)	5 (9.3)	7 (6.3)	3 (3.8)	4 (7.7)

Of those who developed ductal or colloid carcinoma, there was no difference in overall, systemic or locoregional recurrence between precursor subtypes (*p* > 0.05).

### Survival Outcomes

The median length of survival for gastric, pancreatobiliary, intestinal and mixed subtypes was 41, 43, 50 and 31 months, respectively. There was no significant difference in survival between precursor subtypes in the overall cohort or in ductal or colloid carcinoma subgroups.

### Impact of Adjuvant Chemotherapy

Adjuvant chemotherapy did not reduce the recurrence rate in any precursor epithelial subtype (gastric, logrannk, *p* = 0.134; pancreatobiliary, *p* = 0.442; intestinal, *p* = 0.546; mixed, *p* = 0.613), this was also demonstrated in multivariate CPHMs (*p* = 0.914; *p* = 0.385; *p* = 0.381; *p* = 0.551, respectively)

**Fig. 2:**
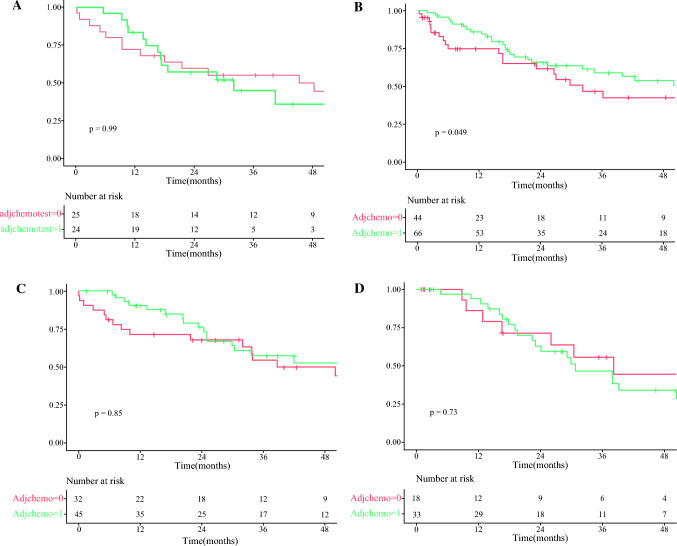
Overall survival with and without adjuvant chemotherapy in (**A**) gastric, (**B**) pancreatobiliary, (**C**) intestinal and (**D**) mixed precursor epithelial subtype

Adjuvant chemotherapy improved overall survival in pancreatobiliary subtype (*p* = 0.049) but not in gastric (*p* = 0.992), intestinal (*p* = 0.852) or mixed subtype (*p* = 0.723; Fig. 2). In multivariate analysis, adjuvant chemotherapy was positively associated with improved overall survival in pancreatobiliary, albeit with borderline significance (*p* = 0.058; Supplementary Figs. 1–4).

No precursor epithelial subtype had a superior survival response (gastric, *p* = 0.845; pancreatobiliary, *p* = 0.214; intestinal, *p* = 0.378, mixed, *p* = 0.302) or reduction in overall recurrence (gastric, *p* = 0.841; pancreatobiliary, *p* = 0.642; intestinal, *p* = 0.800, mixed, *p* = 0.601) with any particular chemotherapy regimen (GEM, GEM-CAP, FFX, other).

## Discussion

To the best of our knowledge, the present study is the largest to date reporting clinicopathological features, recurrence patterns and survival outcomes of A-IPMN subtypes and the first study to compare the response to adjuvant chemotherapy in different epithelial subtypes. Key differences in clinicopathological features were reported which help explain the inferior outcomes in gastric, pancreatobiliary and mixed subtypes compared with intestinal subtype. Gastric, pancreatobiliary and mixed subtypes were found to have comparable clinicopathological features and long-term recurrence and survival outcomes (median follow-up 6 years, 5 months). Pancreatobiliary subtype was found to have a superior survival with adjuvant chemotherapy.

Previous studies have investigated the types of precursor subtypes amongst A-IPMNs.^19^ Kim et al. found that the majority of A-IPMN were of pancreatobiliary subtype and similarly, Kang et al. found that proportions of invasive IPMN were highest in pancreatobiliary subtypes (57.9%).^4,20^ This contrasts with gastric subtypes which according to Nakata et al. are malignant in 14.1%. The metanalysis performed by Koh et al. found that gastric subtype was associated with a lower likelihood of tumour invasion compared with other subtypes.^21^ Similarly, the present data finds gastric and pancreatobiliary subtype in 18.4% and 37.4% of A-IPMNs, respectively, but demonstrates comparable rates of clinicopathological characteristics (e.g. differentiation and lymph node involvement) between the two subtypes.

Previous studies have demonstrated that non-intestinal IPMN subtypes were positively associated with lymphatic invasion and tubular invasion (*p* < 0.05) compared with intestinal subtypes.^22,23^ This is consistent with the present findings, where both gastric and pancreatobiliary subtypes were positively associated with ductal carcinomas and lymph node involvement. In addition, gastric and pancreatobiliary subtypes are more advanced tumours at resection as per the AJCC stage and are more likely to originate from a branch duct. The association between mixed subtype and both higher rates of poor differentiation and ductal invasion was also demonstrated.

Nakata et al. found that intestinal subtype A-IPMNs have superior survival compared with non-intestinal subtype A-IPMNs.^23^ Sadakari et al. report a 0% 5-year survival rate in non-intestinal subtypes across their 30-patient cohort, and Yamada et al. report a lower 5-year survival in non-intestinal compared with intestinal-type (52.7% versus 89.7%; *p* = 0.030; *n* = 56).^22,24^ The present results confirm low 5-year survival rates in both gastric (21.3%) and pancreatobiliary (35.4%) subtypes, with the advantage of a larger cohort. Whilst it is evident that intestinal subtypes have superior outcomes compared with other subtypes, less well investigated is the relationship between gastric, pancreatobiliary and mixed subtypes. Our findings demonstrate similar long term recurrence rates and survival outcomes between gastric, pancreatobiliary and mixed subtypes.

It is well established that both the epithelial subtype of the precursor lesion determines the invasive component.^5,25^ The metanalysis performed by Koh et al. find that intestinal subtype had the greatest propensity for colloid carcinoma.^21^ Interestingly, the present results find that not all A-IPMNs derived from intestinal subtypes will have a colloid carcinoma and a small yet unexpected proportion of gastric (6.3%) and pancreatobiliary (9.3%) subtypes developed an associated colloid carcinoma.

Mino-Kenudson M et al. concluded in 61 invasive IPMNs that gastric subtypes were associated with a worse prognosis because of their association with the more aggressive ductal carcinoma.^25^ Kang et al. also found that the prognostic value of precursor epithelial subtype was removed after accounting for other relevant clinicopathological variables.^4^ In the present study, when investigating ductal and colloid carcinomas individually, the precursor epithelial subtype did not influence recurrence or survival in the present study. Intestinal subtypes that developed ductal carcinoma also had worse survival than their colloid carcinoma counterparts. This suggests that once malignant, A-IPMN outcome is more closely associated with the invasive component rather than the precursor epithelial subtypes.

The Fukuoka consensus statement and the American College of Gastroenterologists Clinical Guidelines make no recommendations on the role of adjuvant chemotherapy.^10,26–28^ The recently updated Kyoto guidelines report that the role of adjuvant chemotherapy in resectable disease is unknown owing to a lack of high-quality evidence.^29^ The data that supports adjuvant chemotherapy regimens in pancreatic cancer are derived from patients with PDAC.^30–32^ The studies that demonstrate improved outcomes with adjuvant chemotherapy in A-IPMNs are small cohort studies and results are conflicting.^11–13,33^ The impact of precursor epithelial subtype on response to adjuvant chemotherapy has not been investigated, to the best of our knowledge.

The present study finds an improved survival in patients with pancreatobiliary subtype who receive adjuvant chemotherapy. Importantly, this analysis was consistent once adjusting for established high-risk characteristics (e.g. R1 resection differentiation, lymph node status) to account for selection bias.^11,12,28,34,35^ Interestingly, no improvement in recurrence was observed but this could be secondary to differing follow-up protocols between institutions and undiagnosed recurrence. It is feasible that the heterogeneous immunohistochemical and morphological nature of A-IPMN precursor epithelial subtypes influences the impact of adjuvant chemotherapy.^13,36^ In ampullary adenocarcinoma, despite inferior prognosis, pancreatobiliary subtype has also demonstrated superior response to gemcitabine-based adjuvant chemotherapy regimens compared with intestinal subtypes. Similarities in molecular phenotypes and mutational status between pancreatobiliary subtype of ampullary adenocarcinoma, pancreatobiliary A-IPMNs and PDAC may explain the superior response to adjuvant chemotherapy in these subgroups.^37–40^ Further research should investigate this finding to determine the most appropriate adjuvant chemotherapy regimens for each subtype.

Although this is the largest study to date that investigates precursor epithelial subtype in invasive patients, the sample size was a limitation. A significant proportion of patients had pancreatobiliary subtype with fewer patients with gastric and intestinal subtype. A further limitation is the potential for modest interobserver agreement rate between even expert histopathologists in typing intraductal precursor lesions. A-IPMNs are relatively rare, making it difficult for histopathologists to gain substantial exposure and experience with this entity. Accurate morphological distinction between IPMN subtypes may represent a challenge to histopathologists, especially if immunohistochemical panels for subtyping these lesions are not available.

In conclusion, the present study reports clinicopathological features, recurrence and survival outcomes, as well as response to adjuvant chemotherapy in precursor epithelial subtypes. Key differences in clinicopathological features were reported which explain the inferior outcomes in gastric, pancreatobiliary and mixed subtypes compared with intestinal subtype. Gastric and pancreatobiliary subtypes were found to have inferior outcomes likely explained by their association with ductal carcinoma. Pancreatobiliary subtype was found to have improved survival with adjuvant chemotherapy.
